# Untargeted Metabolomic Analysis Combined With Multivariate Statistics Reveal Distinct Metabolic Changes in GPR40 Agonist-Treated Animals Related to Bile Acid Metabolism

**DOI:** 10.3389/fmolb.2020.598369

**Published:** 2021-01-15

**Authors:** Hannes Doerfler, Dana-Adriana Botesteanu, Stefan Blech, Ralf Laux

**Affiliations:** ^1^Department of Drug Metabolism & Pharmacokinetics, Boehringer Ingelheim Pharma GmbH & Co. KG, Biberach, Germany; ^2^Department of Drug Discovery Sciences, Boehringer Ingelheim RCV GmbH & Co KG, Vienna, Austria

**Keywords:** metabolomics, drug safety—clinical pharmacology, ion mobility—mass spectrometry, OPLS DA, GPR40 agonists

## Abstract

Metabolomics has been increasingly applied to biomarker discovery, as untargeted metabolic profiling represents a powerful exploratory tool for identifying causal links between biomarkers and disease phenotypes. In the present work, we used untargeted metabolomics to investigate plasma specimens of rats, dogs, and mice treated with small-molecule drugs designed for improved glycemic control of type 2 diabetes mellitus patients via activation of GPR40. The *in vivo* pharmacology of GPR40 is not yet fully understood. Compounds targeting this receptor have been found to induce drug-induced liver injury (DILI). Metabolomic analysis facilitating an integrated UPLC-TWIMS-HRMS platform was used to detect metabolic differences between treated and non-treated animals within two 4-week toxicity studies in rat and dog, and one 2-week toxicity study in mouse. Multivariate statistics of untargeted metabolomics data subsequently revealed the presence of several significantly upregulated endogenous compounds in the treated animals whose plasma level is known to be affected during DILI. A specific bile acid metabolite useful as endogenous probe for drug–drug interaction studies was identified (chenodeoxycholic acid-24 glucuronide), as well as a metabolic precursor indicative of acidic bile acid biosynthesis (7α-hydroxy-3-oxo-4-cholestenoic acid). These results correlate with typical liver toxicity parameters on the individual level.

## Introduction

The metabolome is defined as the collection of all small-molecule metabolites circulating in an organism (Fiehn, 2002). Metabolomics is an emerging science aimed at identifying and quantifying all small molecules present in a complex biological sample present at a specific time point (Goodacre et al., 2004).

Since changes in the metabolome level are caused by both environmental and biological circumstances, the investigation of the metabolome has the potential to provide insight into the genotype–phenotype relationship of an organism (Fiehn, 2002; Schuhmacher et al., 2013). Thus, by their untargeted nature, metabolomic analysis techniques are a promising approach for creating novel insights into physiological mechanisms via unfolding the biochemical composition of complex biogenic samples (Weckwerth and Morgenthal, 2005).

The method of choice for metabolomic analyses is chromatography coupled to mass spectrometry, granting both sensitivity and low detection limits (Dettmer et al., 2007). Metabolomic techniques, however, remain limited in their degree of reliance concerning the identification of detected signals within an untargeted metabolomics study. Metabolomic research historically emerged using gas chromatography coupled to mass spectrometry (GC-MS) and has become a gold standard for identifying and quantifying low-molecular compounds up to about 600 Da through the development of common protocols and the creation of spectral databases. This allows for the day-to-day identification of core compound families like sugars and amino acids (Fiehn, 2008, 2016; Kind et al., 2009). To increase the metabolic coverage to compounds that exceed the limitations of GC-MS, intense efforts are being made to conduct metabolomic analyses on the LC-MS (liquid chromatography–mass spectrometry) platform, which can cover a wider range of potentially interesting biogenic molecules (Halket et al., 2005). LC-MS based metabolomics, however, still lacks standardization and comprehensive databases for reliable metabolite identification, due to the inherent complexity of metabolites that do not follow a common building block like proteins.

We note that initiatives to standardize output from LC-MS-based metabolomic analyses have been initiated (Members et al., 2007), and databases are constantly being created and augmented (Smith et al., 2005; Horai et al., 2010; Wishart et al., 2017). However, up to date, a robust validation by a reference standard concerning retention time and fragment spectrum match is needed for the reliable identification of a metabolite (Schrimpe-Rutledge et al., 2016).

Despite these technological challenges, untargeted metabolic screening facilitating liquid chromatography hyphenated to high-resolution mass spectrometry is already recognized as an explorative bioanalytical tool in various areas of life sciences (Gertsman and Barshop, 2018). In the field of biomarker research and discovery, untargeted metabolomics is expected to yield novel insights into biochemical pathways and ultimately lead to improved understanding of the biological system at hand (Monteiro et al., 2013; Zhao and Lin, 2014).

In this study, we employ an integrated approach of UPLC coupled to traveling-wave ion mobility separation (TWIMS)-TOF–mass spectrometry with subsequent multivariate analysis to investigate endogenous compounds in toxicity studies performed with GPR40 agonist-type drugs. TWIMS is a separation technique in the gas phase, which discriminates molecules due to their three-dimensional shape. In combination with UPLC-MS, this setup introduces the drift time—more commonly expressed as collisional cross section (CCS, in Å^2^)—as additional separation parameter besides mass-to-charge (m/z) ratio, retention time, and intensity (Shvartsburg and Smith, 2008). The determination of CCS values is especially helpful to distinguishing co-eluting compounds of the same m/z ratio, but different steric properties (Santos et al., 2010; Rainville et al., 2017). We have previously shown that this mass spectrometric platform is well-suited to investigate small molecules in a complex biological sample in drug metabolism studies (Blech and Laux, 2013; Fiebig et al., 2016); for the present untargeted metabolomics study, the full potential of described UPLC-TWIMS-HRMS facility is harnessed as well.

GPR40, also known as free fatty acid receptor 1 (FFAR1), is a class A G-protein coupled receptor mainly expressed in pancreatic beta cells and proven to modulate glucose-dependent insulin secretion over short- and medium-chain fatty acids (Briscoe et al., 2003; Itoh et al., 2003). In recent years, this receptor has attracted much attention in pharmacological research as it can be triggered by synthetic small-molecule agonists investigated for the potential treatment for diabetes mellitus type 2 (Burant, 2013; Poitout and Lin, 2013). In 2010, fasiglifam was reported as the first drug to selectively stimulate GPR40 and significantly improve glycemic control, making it a promising candidate drug for patients with type 2 diabetes (Tsujihata et al., 2011). However, in 2015, development of fasiglifam was terminated in phase III clinical trials due to safety concerns regarding liver toxicity (Li et al., 2015).

In the present work, we investigated the effects of two novel synthetic GPR40 agonists in a 4-week rat and dog and a 2-week mouse toxicity study with compound BI-1 and BI-2, respectively. We aimed to obtain a deeper understanding of mechanistic and toxic implications of GPR40 activation. Untargeted metabolic screening in plasma samples in combination with a specialized multivariate statistical approach revealed the presence of seven upregulated bile acids, similar to reported findings on toxicologically relevant cholestatic activity known to be induced by GPR40 agonists (Li et al., 2015). Additionally, a specific bile acid precursor, 7α-hydroxy-3-oxo-4-cholestenoic acid (7-HOCA), was present in the treated plasma, which revealed insights into the biosynthetic route of bile acids in the performed studies. Furthermore, a glucuronide metabolite of chenodeoxycholic acid (CDCA-24G) was found, which was recently described as a substrate of organic anion transporters (Takehara et al., 2017). Further efforts to establish causal links between unbiased metabolomics methods and disease phenotypes will be crucial for increasing our understanding of disease pathology.

## Results

### Visualization of Discriminating Metabolites Between Control and Treatment Groups

Extracted m/z features aligned by mass, retention, and drift time were assembled in a data matrix together with their respective chromatographic injections for subsequent statistical analysis. This resulted in a data matrix X, containing N observation rows (injections) and K variable columns (m/z features), where each observation is characterized by hundreds to thousands of m/z values (Supplementary Figure 1). Such data sets represent a so-called megavariate analytical problem featuring multiple latent variables (Eriksson et al., 2013), which can be approached by different kinds of multivariate data analysis (Rubingh et al., 2006; Eriksson et al., 2013; Worley and Powers, 2013). In our setup, we used OPLS-DA (orthogonal partial least squares discriminant analysis) to investigate the data. OPLS-DA is especially well-suited to highlight discriminating variables in a two-class problem; thus, the method of choice to investigate factors that cause group separation between two conditions in multidimensional data sets (Trygg and Wold, 2002; Eriksson et al., 2013)—in our case the comparison of control vs. treatment animals. The most important visualization of an OPLS-DA model helpful in identifying discriminating variables is the so-called S-Plot. The S-Plot is a scatter plot which visualizes variable influence in a model (Wiklund et al., 2008).

**Figure 1:**
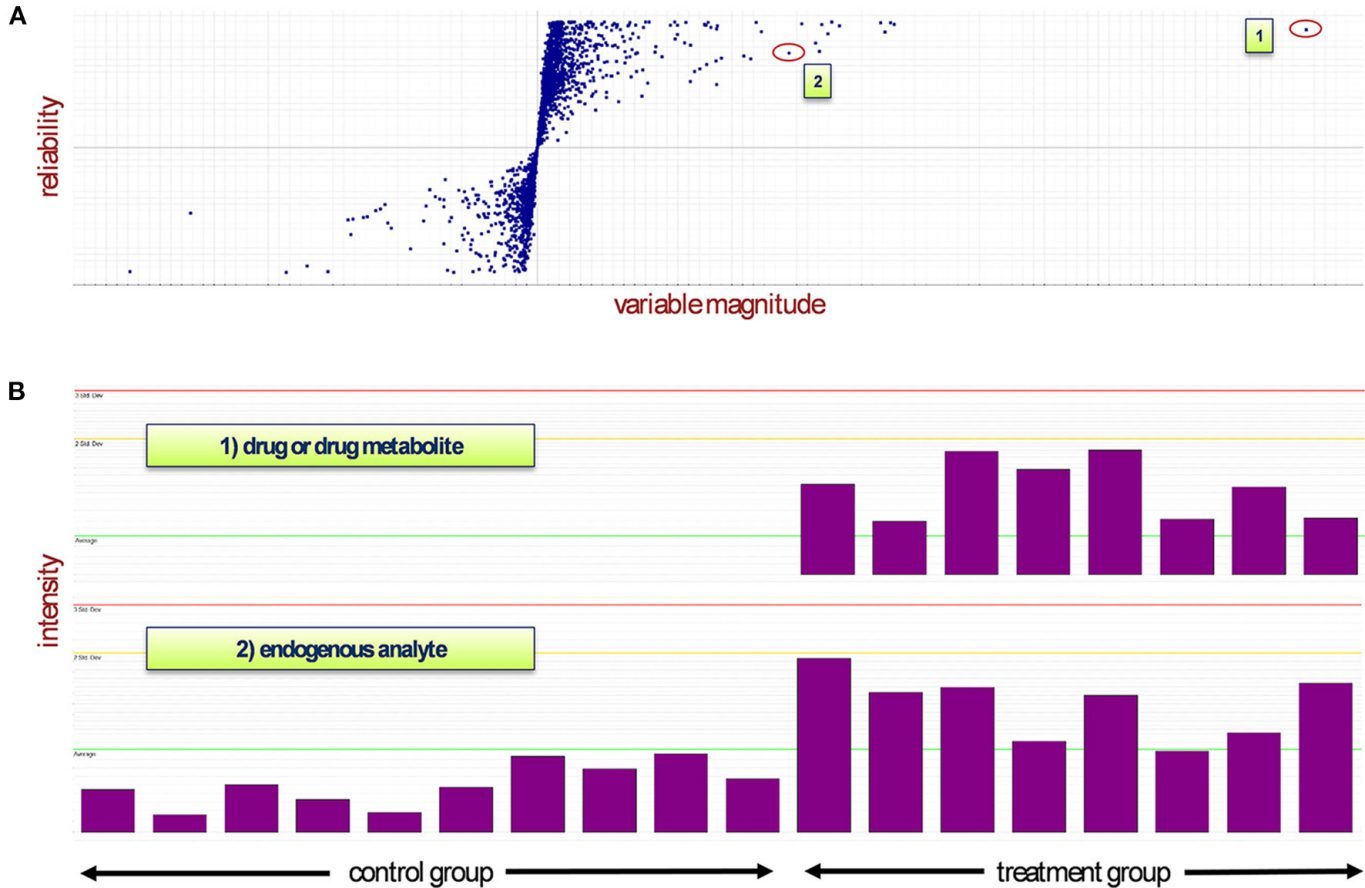
**(A)** The S-Plot distributes the variables between two groups depending on their abundance in each sample (observations). The treatment group is represented in quadrant 1 while the control group lies in quadrant 3. **(B)** Mass spectrometry signals of two different types of analyte: Case 1 represents a drug or drug metabolite, which will appear near the top of the S-Plot due to its non-occurrence in the control samples (highest reliability and high magnitude). In case 2, depending on their presence in control and treatment groups, endogenous compounds will appear top-right or bottom-left in the S-Plot. An ideal biomarker has both high reliability and high magnitude and thus would be located in either one of the corners of the two groups. Red line: triple standard deviation; yellow line: double standard deviation; green line: average.

In the present study, we used the S-Plot to spot the most prominently changing chemical entities between control and treatment groups. Figure 1 shows a typical S-Plot resulting from comparing a control (non-treatment) group of 10 animals against a dose group consisting of eight animals. S-Plots depict variable magnitude (modeled co-variation) on the x-axis and reliability (modeled correlation) on the y-axis. For experiments that aim to identify molecules with biomarker traits—as is often the case in metabolomics studies—both high variable magnitude and correlation are desired, thus compounds of interest are spotted in the top-right corner in case of upregulation (and bottom-left corner in case of downregulation). If xenobiotics (as in our case, the drug and its metabolites) are present in the sample and captured by the analysis, they will be very visible in the S-Plot, because these molecules are not present in the non-treated group and thus cause maximum variation between control and treatment and thus are located near the top of the correlation axis. This effect can be exploited to quickly identify drug metabolites in drug metabolism studies, whose aim is the detection and structure elucidation of the major biochemical modifications of a drug after administration.

In our three studies, each S-Plot consisted of roughly 3,000 m/z features. In order to reduce this amount of “known unknown” analytes toward a tangible set of compounds of potential physiological interest, only m/z features appearing in at least two or our three studies within the range of 0.1 or higher co-variation and 0.4 or higher correlation were kept in the data set.

### Identification of Specific Bile Acids as Significantly Upregulated Compounds

**Figure 2:**
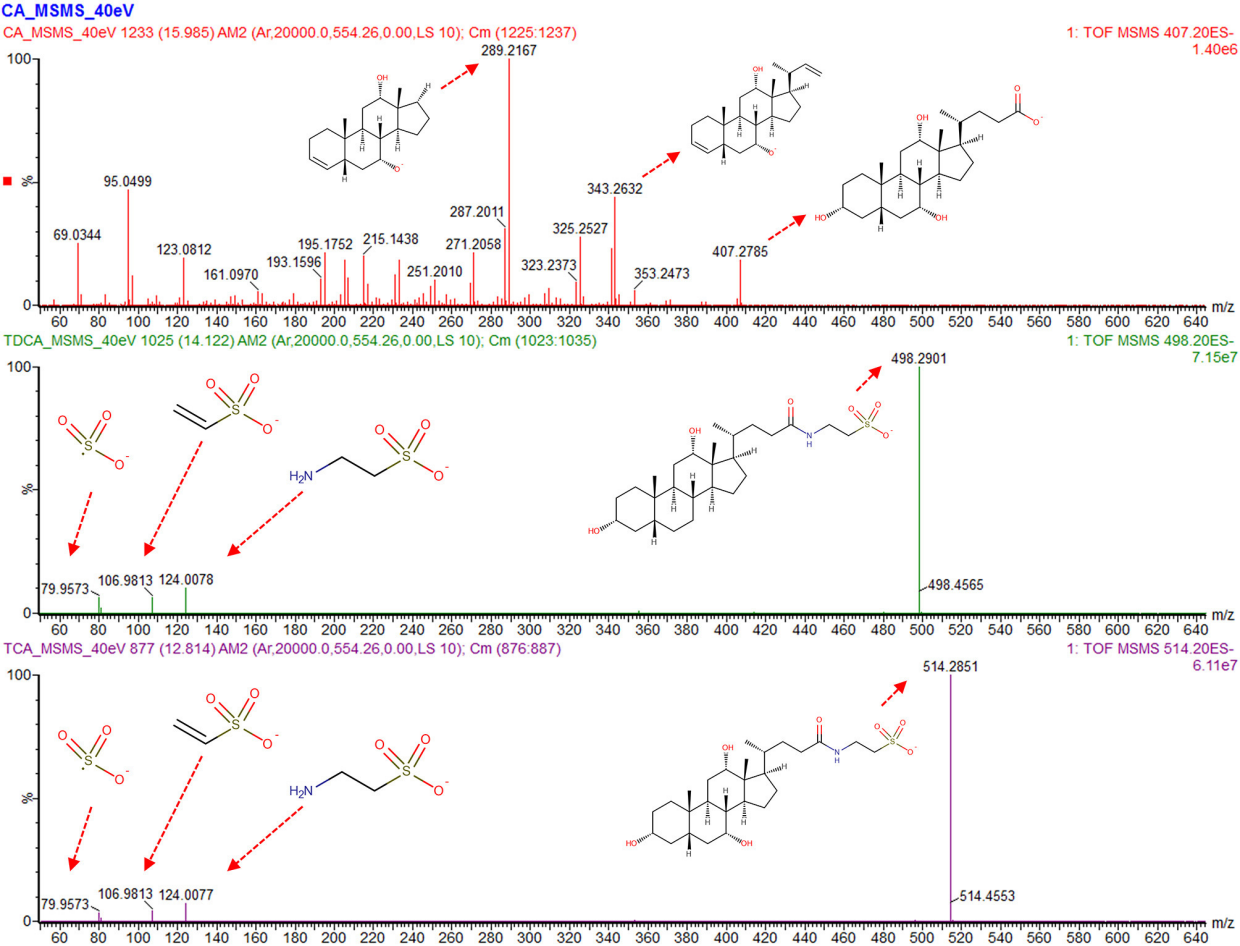
Analysis of the fragmentation (“high”) spectra of the bile acids which display the most significant changes between control and dosed groups. Main peaks for cholic acid (CA) contain decarboxylation combined with dehydration and desaturation (m/z 343.26) and further cleavage on position 17 (m/z 289.21). For taurodeoxycholic acid (TDCA) and taurocholic acid (TCA), the taurine peaks (m/z 124), [taurine-NH3], 106.98, as well as the sulfur trioxide radical anion (m/z 79.95) are visible, with a mass accuracy of each fragment below 5 mDa to between the compound in the sample and purchased reference compound.

Systematic evaluation of the S-Plots of the three studies unfolded the presence of several upregulated m/z features in the sectors of the treated group. With the fragmentation (“high”) spectrum being in accordance in all three studies each (rat, dog, mouse), exhaustive structure annotation by interpretation of fragment spectra and literature search was done, which resulted in the identification of a set of compounds belonging to the bile acid family. All identified compounds were validated by reference standards (see Figures 2, 4 and Supplementary Figures 2–4).

Table 1 shows all detected bile acids that could be verified via purchasable reference substances. In particular, cholic acid (CA, m/z 407.28 [M-H]^−^), taurodeoxycholic acid (TDCA, m/z 498.29 [M-H]^−^), and taurocholic acid (TCA, m/z 514.28 [M-H]^−^) were found to be present in significantly elevated levels in the high-dose group of the dog and rat study. Figure 2 shows an annotated fragmentation (“high”) spectrum of these identified bile acids. Subsequently, individual responses (peak area) for each identified compound were extracted from the data and compared within each study. In a similar fashion, two bile acid-related compounds could be elucidated from the data set via the described untargeted approach due to upregulation in all the high-dose groups, namely, 7α-hydroxy-3-oxo-4-cholestenoic acid (7-HOCA) and chenodeoxycholic acid glucuronide (CDCA-24G).

**Table 1:** Overview of identified bile acids and bile acid-related compounds in the three toxicity studies.

**Chemical name**	**Abbreviation**	**Molecular formula**	**Monoisotopic mass**	**[M–H]^**−**^**	**CCS [M–H]^**−**^**	**Main fragments**	**CAS number**	**Mass accuracy (mDa)**
Cholic acid	CA	C_24_H_40_O_5_	408.288	407.280	196.80	289, 343	81-25-4	0.4
Taurodeoxycholic acid	TDCA	C_26_H_45_NO_6_S	499.297	498.290	200.40	79, 106, 124	516-50-7	1.3
Taurocholic acid	TCA	C_26_H_45_NO_7_S	515.292	514.284	201.43	79, 106, 124	81-24-3	0.7
Deoxycholic acid	DCA	C_24_H_40_O_4_	392.293	391.285	198.90	343, 69	83-44-3	2.1
Glycocholic acid	GCA	C_26_H_43_NO_6_	465.309	464.302	195.58	400, 74	475-31-0	0.5
Glycodeoxycholic acid	GDCA	C_26_H_43_NO_5_	449.314	448.307	193.20	402, 74	360-65-6	0.7
Hyodeoxycholic acid	HDCA	C_24_H_40_O_4_	392.293	391.285	203.37	373, 69	83-49-8	1.1
7α-Hydroxy-3-oxo-4-cholestenoic acid	7-HOCA	C_27_H_42_O_4_	430.308	429.301	198.73	411, 123	115538-85-7	1.3
Chenodeoxycholic acid 24-glucuronide	CDCA-24G	C_30_H_48_O_10_	568.325	567.317	220.46	391, 113, 85	208038-27-1	0.8

**Figure 3:**
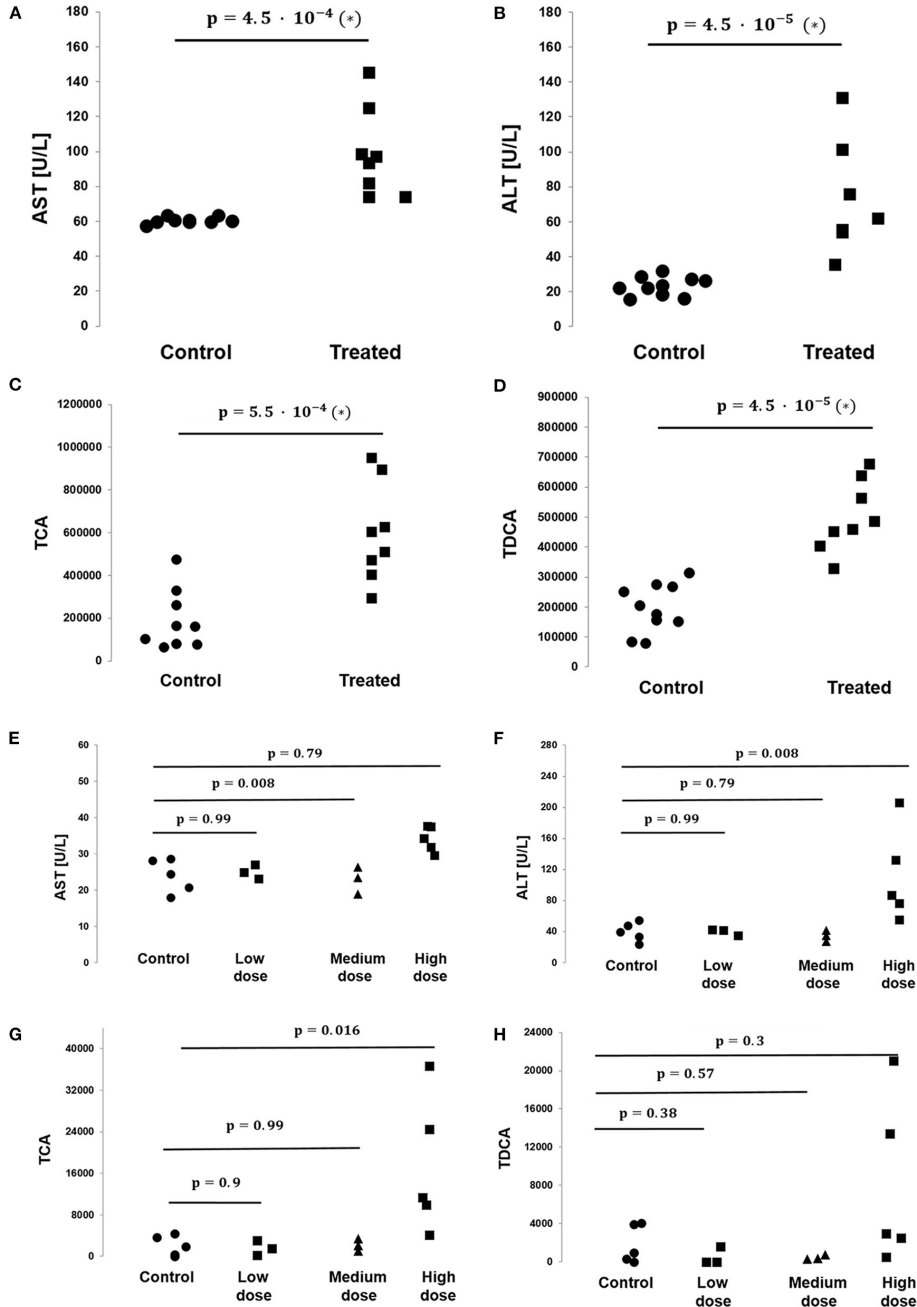
Levels of bile acids TCA and TDCA and the toxicologically relevant liver enzymes AST and ALT as measured in the rat and dog studies. **(A)** AST levels are significantly elevated in the treated vs. control group animals in the rat study (*N* = 10 vs. *N* = 8, respectively, *p*-value = 4.5 · 10^−4^, non-parametric Mann–Whitney test). **(B)** ALT levels are significantly elevated in the treated vs. control group animals in the rat study (*N* = 10 vs. *N* = 8, respectively, *p*-value = 4.5 · 10^−5^, non-parametric Mann–Whitney test). **(C)** TCA levels are significantly elevated in the treated vs. control group animals in the rat study (*N* = 10 vs. *N* = 8, respectively, *p*-value = 4.5 · 10^−5^, non-parametric Mann–Whitney test). **(D)** TDCA levels are significantly elevated in the treated vs. control group animals in the rat study (*N* = 10 vs. *N* = 8, respectively, *p*-value = 4.5 · 10^−5^, non-parametric Mann–Whitney test). **(E)** No statistically significant difference in AST levels between control and treated groups were found in the BI-1 dog study. Each *p*-value listed and its corresponding horizontal bar refer to the statistical comparison of AST levels between the control and one of the treated groups, either low, medium, or high dose (*N* = 5/3/3/5 for the control/low dose/medium dose/high dose groups, respectively). **(F)** No statistically significant difference in ALT levels between groups was found in the BI-1 dog study. Each *p*-value listed and its corresponding horizontal bar refer to the statistical comparison of ALT levels between the control and one of the treated groups, either low, medium, or high dose (*N* = 5/3/3/5 for the control/low dose/medium dose/high dose groups, respectively). **(G)** No statistically significant difference in TCA levels between groups were found in the BI-1 dog study. Each *p*-value listed and its corresponding horizontal bar refer to the statistical comparison of TCA levels between the control and one of the treated groups, either low, medium or high dose (*N* = 5/3/3/5 for the control/low dose/medium dose/high dose groups, respectively). **(H)** No statistically significant difference in TDCA levels between groups were found in the BI-1 dog study. Each *p*-value listed and its corresponding horizontal bar refer to the statistical comparison of TDCA levels between the control and one of the treated groups, either low, medium, or high dose (*N* = 5/3/3/5 for the control/low dose/medium dose/high dose groups, respectively).

*All compounds listed in the table had detectable levels in all three toxicity studies performed, except for TDCA, which was not detectable in the mouse study in both control and treated samples, and for CA, which was not detectable in the control samples in the mouse and dog studies. Data for the identified bile acids are shown in Figures 3A–H, and the statistical comparisons against the corresponding control groups (where applicable) are described in the text. Mass accuracy of identified compounds is shown exemplary according the values found in the BI-1 dog study*.

### Increased Liver Enzyme Levels Correlate With Increased Plasma Bile Acid Levels in the BI-1 Rat Study

On average, AST, ALT, and ALP enzyme levels in the treated samples were 1.63-, 3.08-, and 1.25- fold higher respectively compared to the levels in the corresponding control samples. AST enzyme levels were strongly and significantly correlated with CA, TDCA, 7-HOCA, and TCA bile acid levels across all samples (Table 2). AST levels were strongly and significantly correlated with ALT levels in all measured samples (Table 3). AST and ALT levels are shown in Figures 3A,B, respectively. Overall, the correlation matrix for ALT, AST, and ALP levels revealed a moderate correlation between these enzyme levels (Table 3).

**Table 2:** Correlation matrix between enzyme and bile acid levels in the rat study (*N* = 18 total samples).

**r (*p*-value)**	**CA**	**TDCA**	**7-HOCA**	**TCA**
AST	0.8 (5e-5)	0.74 (4e-4)	0.9 (4e-7)	0.62 (5.6e-3)
ALP	0.68 (2e-3)	0.47 (4.8e-2)	0.65 (3.5e-3)	0.6 (8.5e-3)
ALT	0.75 (3e-4)	0.64 (4e-3)	0.88 (4e-7)	0.58 (1e-2)

*Pearson's correlation coefficient r and the corresponding p-value for the hypothesis test are reported*.

**Table 3:** Correlation matrix between enzyme levels in the rat study (*N* = 18 total samples).

**r (*p*-value)**	**AST**	**ALP**	**ALT**
AST		0.45 (4e-2)	0.89 (4e-7)
ALP	0.45 (6e-2)		0.49 (4e-2)
ALT	0.89 (4e-7)	0.49 (4e-2)	

*Pearson's correlation coefficient r and the corresponding p-value for the hypothesis test are reported*.

Similarly, for the six bile acid levels under study (CA, TDCA, TCA, GDCA sulfate, CDCA-24G, 7-HOCA), the correlation matrix revealed a strong association between the bile acid levels overall in all measured samples, e.g., CA vs. 7-HOCA, *r* = 0.93, TDCA vs. TCA *r* = 0.88, TCA vs. CA *r* = 0.74. TCA and TDCA levels are shown in Figures 3C,D, respectively.

On average, CA, TDCA, TCA, and 7-HOCA bile acid levels in the treated samples were 14.21-, 2.56-, 3.29-, and 9.15-fold higher, respectively, compared to the levels in the corresponding control samples. Of note, GDCA sulfate and CDCA-24G were absent in all the control animals in this study (all measured values were 0), while in the treated animals, GDCA sulfate levels were measured at 2796.18 ± 2129.41 (mean ± SD), and CDCA-24G at 70739 ± 105413.64 (mean ± SD).

### Liver Enzyme Levels and Plasma Bile Acid Levels in the BI-1 Dog and BI-2 Mouse Studies

In the dog study, AST enzyme levels in the low-, medium-, and high-dose groups were, on average, 1.04-, 0.96-, and 1.42-fold, respectively, compared to the levels in the control samples. ALT levels were, on average, 1.003-, 0.86-, and 2.8-fold in the low-, medium-, and high-dose groups compared to the control sample levels. ALP levels were, on average, 0.85-, 0.78-, and 4.52-fold in the low-, medium-, and high-dose groups compared to the control sample levels.

AST enzyme levels were strongly and significantly correlated with TCA bile acid levels across samples in the dog study (Table 4). AST levels were representative of other measured liver enzyme parameters, specifically ALT and ALP levels (AST and ALT levels shown in Figures 3E,F, respectively). The correlation matrix for ALT, AST, and ALP levels revealed a strong correlation between these enzyme levels overall (Table 5).

**Table 4:** Correlation matrix between enzyme and bile acid levels in the dog study (*N* = 16 total samples).

**rho (*p*-value)**	**TDCA**	**TCA**
AST	0.65 (6.5e-3)	0.86 (2e-5)
ALP	0.58 (1.8e-2)	0.75 (8e-4)
ALT	0.5 (5e-2)	0.7 (2.5e-3)

*Spearman's correlation coefficient rho and the corresponding p-value for the hypothesis test are reported*.

**Table 5:** Correlation matrix between enzyme levels in the dog study (*N* = 16 total samples).

**rho (*p*-value)**	**AST**	**ALP**	**ALT**
AST		0.69 (2.8e-3)	0.82 (1e-4)
ALP	0.69 (2.8e-3)		0.76 (6.5e-4)
ALT	0.82 (1e-4)	0.76 (6.5e-4)	

*Spearman's correlation coefficient rho and the corresponding p-value for the hypothesis test are reported*.

On average, TDCA bile acid levels in the treated samples were 0.87-, 0.25-, and 4.4-fold in the low-, medium-, and high-dose groups, respectively, compared to the levels in the control samples. Similarly, TCA levels in the treated samples were 0.8-, 1.04-, and 8.4-fold in the low-, medium-, and high-dose groups, respectively, compared to the levels in the control samples. TCA and TDCA levels are shown in Figures 3G,H, respectively. Of note, CA was absent in all the control animals in this study (all measured values were 0), while in the treated animals, CA levels were measured at 767 ± 783, 896 ± 751.4, and 12550 ± 783 (mean ± SD) in the low-, medium-, and high-dose groups, respectively.

We note that the small sample sizes in the low- and medium-dose groups in the dog study prevent the statistical analysis from being extrapolated to the overall treated population. As a result, the comparative and correlative findings should be interpreted with caution and viewed as hypothesis-generating because of the small number of samples we assessed.

In the mouse study, AST, ALT, and ALP enzyme levels in the treated samples were, on average, 1.16-, 1.53-, and 0.77-fold, respectively, compared to the levels in the corresponding control samples (see Supplementary Figure 6). Since TDCA levels were not detectable in either control or treated mouse samples, and CA levels were not detectable in the control mouse samples, we did not perform a formal statistical comparison between the control and treated groups on bile acid levels and we did not assess the correlation between liver enzyme and bile acid levels.

### Identification of Single Bile Acids in Untreated Plasma Samples

**Figure 4:**
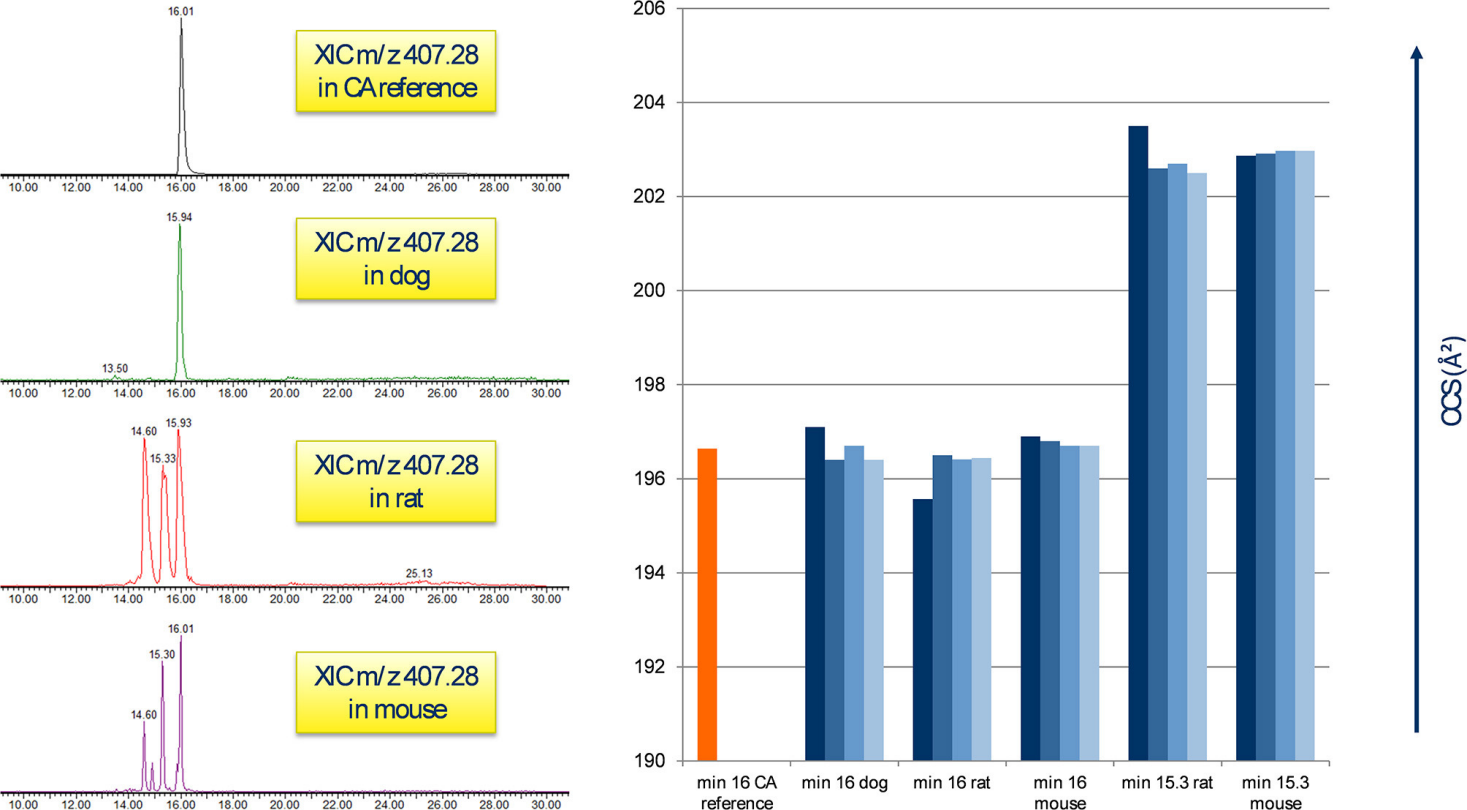
Appearance of CA in untreated plasma samples. **Left**: the reference peak of the CA standard correlates to m/z 407.28 at the minute 16 mark in all three animal species; rodent samples feature additional peaks at min 15.3 and 14.6. **Right**: comparison of CA standard CCS (min 16) with the peaks of each 4 biological at minute 16 and 15.3. A clear difference in the CCS value between the signals is visible, probably by the presence of isomeric forms of CA in raw rodent plasma.

Identification of single bile acids from plasma was hampered due to the presence of additional peaks corresponding to the same precursor value of one bile acid. It has been reported that bile acids are prone to epimerization caused by the microbial intestine, leading to different conformational modifications of the original structure while maintaining the same mass (Aldini et al., 1989; Rudling, 2016). Extraction of ions with m/z 407.28 from the chromatograms, which corresponds to the [M-H]^−^ of CA, yielded a single peak in the reference standard of CA, as well as a single peak in the treated specimen of the dog study at the minute 16 mark, with both peaks displaying a CCS value of 196 Å^2^ as depicted in Figure 4; in the treated rat and mouse specimen, several signals of m/z 407.28 could be observed additional to the one at minute 16, predominantly at minute 15.3 and 14.6, with a CCS value of 203 Å^2^ (Figure 4). The additional peaks in the rodent studies, however, showed a near-exact fragment spectrum compared to the reference compound and the compound in the dog study, while featuring different CCS values. The same behavior was observed with TDCA and TCA (not shown). This leads us to assume the additional peaks are isomeric forms of said bile acids in the rodent plasma, which have undergone epimerization at some point.

## Discussion

### Physiological Implications of Increased Plasma Bile Acids

Bile acids have been studied extensively in the past, as these metabolites perform a number of vital physiological functions (Marin et al., 2015). Collectively, they form a family of steroid acids and are synthesized via a multistep pathway starting from cholesterol in the liver. Upon conjugation to glycine or taurine, a process through which the bile acids become cell-impermeable, they are stored as bile salts in the gallbladder, from which they are secreted into the intestinal tract, namely, as bile flow. In the terminal ileum, they are reabsorbed by bile-salt transporters, which in return inhibits bile acid synthesis (Monte et al., 2009).

Bile acids are water-soluble compounds whose best-known role is to aid in the absorption of lipid nutrients through the formation of mixed micelles. Among their other functions, they are also involved in biliary secretion of toxins (Amigo et al., 2003) and can act as antimicrobial agents in the gut (Ridlon et al., 2014). In recent years, bile acids have been identified as important signaling molecules for endocrine processes (Monte et al., 2009; Chiang, 2013; Schadt et al., 2016).

A possible explanation for the elevated bile acid levels in treatment plasma samples is the emergence of cholestatic effects (Zamek-Gliszczynski et al., 2012). Cholestasis is a condition where bile flow from the liver to the intestine tract is impaired, with accumulation of bile acids in the liver and decreased bile in the intestine. Bile acids in abnormally high concentrations damage the bile duct epithelial cells and hepatocytes, while chronic cholestasis eventually leads to inflammation and ultimately liver failure (Li and Apte, 2015).

An important factor involved in the hepatic excretion of xenobiotics with regard to drug metabolism is the bile salt export pump (BSEP). BSEP is an ABC (ATP-binding cassette)-transporter located in the hepatic canalicular membrane and is responsible for the secretion of bile acids into bile in humans. Mutations in BSEP or disturbances in its homeostasis can result in toxic bile salt accumulation (Kubitz et al., 2012). Stieger et al. showed that certain drugs can negatively affect BSEP and thus induce acquired cholestasis, which represents an example of drug-induced liver injury (DILI) (Stieger et al., 2000; Stieger, 2010).

One of the first examples of reduced bile secretion caused by the administration of a drug was troglitazone, which was found to competitively inhibit human and dog BSEP (Preininger et al., 1999; Stieger, 2010). This mechanism has been reported in the more recent case of the GPR40 agonist fasiglifam in 2015, whose development was terminated during clinical phase III due to concerns about liver toxicity: fasiglifam increased CA, TDCA, TCA, and UDCA levels by at least 2 times (Li et al., 2015). With the exception of UDCA, which was not observed in our studies, these findings align with our results and suggest a similar mechanism of BI-1 and BI-2 in the enterohepatic system, which could eventually lead to drug-induced liver injury. This is supported by clinical pathological analysis of BI-1 and BI-2 regarding the upregulation of the liver enzymes AST, ALT, ALP, GGT, and GLDH, as well as total and direct bilirubin and total bile acid increase (data not shown). While total bile acid is a useful parameter to monitor liver function, it is non-specific, and determination of single bile acid profiles by liquid chromatography and mass spectrometry for better mechanistic understanding of clinically relevant data have been proposed before (Parraga and Kaneko, 1985; Ducroq et al., 2010; Cepa et al., 2018). We found a clear correlation between individual bile acid and liver enzyme levels, suggesting that single plasma bile acids might be a sensitive marker for cholestatic effects. For example, a high correlation coefficient between TCA and AST levels in the rat study with BI-1 suggests that TCA mirrors AST levels and may be used as an additional potential toxicological parameter in drug safety studies (Figure 4).

Another bile acid-related compound, CDCA glucuronide, could be identified in our rat and dog studies, specifically in the high-dose groups compared to the control individuals. While CDCA can be glucuronidated preferably in position 3 and 24, fragmentation spectra of the available CDCA-24G reference compound were in full accordance with the spectra of the [M-H]^−^ precursors in the plasma samples, pointing to the actual presence of this acyl glucuronide in our study. Takehara et al. reported CDCA-24G, together with GCDCA-S, as substrates for OATPB1, OATP1B3, and NTCP: uptake of CDCA-24G and GCDCA-S into human hepatocytes was found to be significantly reduced after rifampicin and pioglitazone administration. These results suggest that CDCA-24G and GCDCA-S could be used as surrogate endogenous probes for OATP inhibition, which could in turn be exploited in drug-drug interaction (DDI) studies (Takehara et al., 2017). While GCDCA-S could not be found in our data, CDCA-24G displayed robust signals in the rat and dog studies with BI-1. Further studies are needed to determine whether increased CDCA-24G levels in plasma are a direct result from OATP inhibition by BI-1 or represent a general cholestatic effect.

### 7-HOCA as Biosynthetic Bile Acid Precursor

One intermediate in the acidic route of bile acid synthesis is 7α-hydroxy-3-oxo-4-cholestenoic acid (7-HOCA), which was increased in all of our three animal studies with an average fold change increase of at least five. 7-HOCA can be synthesized extrahepatically and subsequently is taken up by the liver (Bjorkhem et al., 1997). This intermediate has been reported to accumulate in the cerebrospinal fluid in patients with dysfunctional blood brain barrier (Saeed et al., 2014), and in chronic subdural hematoma (Nagata et al., 1992). With respect to its elevated presence in plasma samples, we suspect a treatment-induced activation of the acidic bile acid pathway with the administration of BI-1 and BI-2 in our studies (Shoda et al., 1993). Further studies are needed to determine the physiological implications of elevated 7-HOCA levels during treatment by GPR40 agonists.

### Concluding Remarks

We acknowledge the overall small sample sizes of biological probes available for investigation in the current study. As a result, the comparative and correlative findings should be interpreted with caution and viewed as hypothesis-generating because of the small number of samples we assessed. Our untargeted metabolomics approach revealed the presence of several significantly upregulated endogenous compounds in the treated animals whose plasma level is known to be affected during drug-induced liver injury. Future discovery of causal links between unbiased metabolomics methods and disease phenotypes will be crucial for increasing our understanding of disease pathology.

## Materials and Methods

### Chemicals

BI-1 and BI-2 were synthesized by the medicinal chemistry group of Boehringer Ingelheim Pharma GmbH & Co. KG, Ingelheim, Germany (for structures, see Supplementary Figure 5). Acetonitrile, methanol, water, and formic acid were of analytical grade purity and were purchased from Sigma Aldrich (Steinheim, Germany). CA, TDCA, and TCA reference standards and leucine enkephalin were obtained from Sigma Aldrich (Steinheim, Germany); 7-HOCA was purchased from Avanti Polar Lipids, Inc. (Alabaster, US); CDCA-24G was obtained from Carbosynth (Compton, UK).

### Laboratory Animals

All *in vivo* experiments were conducted through and approved in accordance with institutional guidelines by the local animal welfare officer of Boehringer Ingelheim Pharma GmbH & Co. KG, Biberach, Germany, as well as by the responsible supervisory authority, Tübingen, Germany. Boehringer Ingelheim Pharma GmbH & Co. KG is accredited by the Association for Assessment and Accreditation of Laboratory Animal Care International (AAALAC).

The study conditions were as follows: in the BI-1 toxicity study conducted in rats (stain: Crl:WI(HAN), male), eight animals received a daily oral dose of 1,000 mg/kg; 10 control animals received vehicle only. In the BI-1 toxicity study conducted in male dogs (Marshall Beagle, male), five animals received a daily oral dose of 400 mg/kg of BI-1 (referred to in the main text as high dose); another three animals received 40 and 10 mg/kg of BI-1 (referred to in the main text as medium and low doses, respectively), and five animals received vehicle only. Both toxicity studies were performed for a total duration of 4-weeks each. Vials containing blood samples were collected at the time of necropsy (day 29).

In the BI-2 toxicity study conducted in mice (Crl:CD1(ICR), male), six animals received a daily oral dose of 100 mg/kg of BI-2; an additional nine control animals received vehicle only. This study was performed for a total duration of 2-weeks. Vials containing blood samples were collected at the time of necropsy (day 15).

For all toxicity studies conducted, the doses of compounds BI-1 and BI-2 were chosen according to previous internal toxicity procedures to ensure that in most animals under study a toxicological event is triggered. Two weeks is a common and recommended minimum for performing toxicology studies in rodent or non-rodent specifies in range-finding studies (Derelanko and Hollinger, 2002). Plasma was chosen as the sample collection material as the employment of a trap column followed by an analytical column enabled the direct use of raw plasma; this chromatographic setup allows for minimal sample preparation in order to minimize information loss from the complex sample. The samples analyzed in the current study were collected from either rats, dogs, or mice in different toxicity studies according to sample material availability.

### UPLC-TWIMS-MS^E^ QTOF Analysis

Frozen plasma samples were thawed and centrifuged for 2 min at 8,000 rpm (Sigma 1-15 PK micro centrifuge). A 50-μL plasma sample was conveyed into a micro vial and diluted with 50% solvent A (v:v). Twenty five microliter plasma was subsequently injected into the LC system. Samples were analyzed with a 1D-trap LC system consisting of an Acquity Ultra Performance LC (Binary and Isocratic Solvent Manager), Sample Manager 2777 (Waters) with a trap column (Triart C18-S 10 μm, 20 × 4 mm, YMC), and an analytical column (Triart C18 3 μm, 150 × 3 mm, YMC). Solvent A was 0.02 M ammonium formate + 0.1% formic acid, solvent B was acetonitrile + 0.1% formic acid with the following gradient: 2% B (0 min), 2% B (3 min), 98% B (23 min), 98% B (28 min), 2% B (28.1 min). Flow rate was 0.45 mL min^−1^ with a column temperature of 40°C. The MS-System was a Synapt G2 (Waters) operated on MassLynx 4.1. Samples were measured in negative electrospray mode with the following setting: capillary voltage 1.7 kV, desolvation temperature 450° C; N_2_ flow was 1,000 L h^−1^. For full-scan MS and MS^E^ detection, the quadrupole was set to non-resolving RF-only mode. Ions were subsequently collected in the trap cell and pulsed into the TWIMS (traveling-wave ion mobility separation) cell where analytes were separated according to size/charge ratio (collision cross section, CCS). Maintaining drift separation, analytes were conveyed to the TOF analyzer for exact ion mass measurements. Analytes were subsequently acquired in a mass range of 50–2,000 Da with an alternating collision energy in the transfer cell of 0 (“low spectrum”) and 45 eV (“high spectrum”) with a scan time of 0.5 s. Mass calibration was done in both positive and negative modes in a mass range of 50 to 2,000 Da by injecting a mixture of 100 μL 0.1 M NaOH with 20 μL FA (>98%) in 20 mL ACN/H_2_O 80/20 v:v into the system with a flow rate of 20 μL min^−1^. After mass calibration, the RMS ppm error for exact mass measurements was 0.7 ppm (1.3 mDa). The calibration of the ion mobility module (CCS calibration) was done with a solution containing 50 μg mL^−1^ polyalanine and 5 μg mL^−1^ paracetamol in aqua dest. as reference components with a flow rate of 20 μL min^−1^. CCS calibration was achieved utilizing 14 reference peaks in the range of 230 to 1,150 Da with a residual CCS of below 0.1%. To avoid possible chemical and data interference, internal standards were not used in this study.

### Analysis of Liver Toxicity Parameters

Standard clinical chemistry parameters AST, ALT, ALP, GGT, GLDH, direct bilirubin, and total bilirubin were analyzed on a cobas® 6000 (Roche).

### Data Processing and Statistical Analysis

Recorded data files were transferred to UNIFI (V 1.8.3.116, build 116, Waters) in.raw format and processed according to the following settings: automatic peak detection in a retention time range from 3 to 30 min; high-energy intensity threshold at 50 counts and low-energy intensity threshold at 200 counts. 3D peak detection was enabled using the most intense monoisotopic ion option for quantification and the parameter “area” as response value. Lock mass correction with leucine enkephalin in negative mode was enabled (m/z 554.2615; combine with 3 scans, mass window 0.5 m/z). Negative adducts to be taken into account were –H, +HCOO, +e, +Cl, +CH_3_COO.

For m/z feature creation, mass, retention time, and drift time tolerances were determined over the full range of the chromatogram with the automated setting option in UNIFI. The processed data was subsequently exported as.csv files. Multivariate analysis was carried out in EZInfo (V 3.0.3.0, Umetrics AB). The OPLS/OPLS-DA models were created by labeling the analyzed samples due to sample type (reference/unknown); data were autofitted and Pareto-scaled for model creation. Due to the untargeted nature of this study and high reproducibility of the UPLC-HRMS platform, no normalization was done. The parameters were individually chosen/adapted by the automated peak picking function in EZInfo.

For each individual study, an OPLS-DA model and S-Plot were created; treated vs. control animals constitute the basis for the discriminant analysis in EZInfo. Animals received vehicle only represented the “reference” group in the S-Plot, while animals which received a daily dose of drug treatment represented the “unknown” group.

To determine any statistically significant differences in bile acid and/or liver enzyme levels, we first tested for normality of the data using the Shapiro–Wilk normality test. Data collected in the dog and mouse studies were found to be non-normally distributed. As a result, correlations between bile acid and liver enzyme levels were assessed using the non-parametric Spearman's rank-order correlation, which outputs Spearman's correlation coefficient *rho* and the corresponding *p*-value for the hypothesis test whose null hypothesis states that the two sets of data are uncorrelated. Data collected in the rat study were found to be normally distributed. As a result, correlations between bile acid and liver enzyme levels were assessed using the parametric Pearson's correlation, which outputs Pearson's correlation coefficient *r* and the corresponding *p*-value for the hypothesis test whose null hypothesis states that the two sets of data are uncorrelated. Differences between groups within the same study were assessed using non-parametric Mann–Whitney tests. All statistical analyses were two-tailed. The Holm–Bonferroni correction for multiple comparisons on a single data set was used to calculate sequentially corrected *p*-values, with α = 0.001 set as the determined significance threshold for rejecting the null hypothesis of samples having similarly ranked distributions. All statistical analyses were performed using RStudio version 1.1.442.

### Compound Identification

Annotation of m/z features of interest was done by manual comparison of major fragments found in the literature and in the online database HMDB (Wishart et al., 2017) according to precursor mass within a window of 5 mDa before verification with the purchased reference compound (see Table 1).

All identified compounds were validated by reference standards via level 1 identification (Salek et al., 2013) (see Figures 2, 4 and Supplementary Figures 2–4).

## Summary and Conclusion

We established an untargeted metabolomics platform combining UPLC-MS analysis and high mass accuracy measurements featuring an MS^E^ fragmentation technique with parallel ion mobility spectrometry (IMS). This setup, effectively generating 4-dimensional data sets (m/z, retention time, drift time, intensity), paired with subsequent multivariate statistical analysis (MVA), is suitable for detection of discriminating analytes with biomarker candidates and structural elucidation of unknowns in complex samples and is aimed at refining the mechanistic understanding of drug metabolism and toxicology.

In the present study, we demonstrated the effectiveness of this approach in three toxicity studies performed in rat, dog, and mouse using two internal candidate GPR40 agonists. Herein, various upregulated bile acids (e.g., CA, TDCA, and TCA) in the dosed groups were identified, suggesting the two drugs induced the onset of cholestatic effects. Additionally, two bile acid related compounds (CDCA-24G and 7-HOCA) were found in elevated levels in the collected plasma samples. CDCA-24G was recently reported as surrogate marker for OATP inhibition and its determination could possibly aid in DDI studies in the future (Takehara et al., 2017). Moreover, 7-HOCA is a biosynthetic bile acid precursor, which points to the activation of the acidic pathway of bile acid biosynthesis in this study (Bjorkhem et al., 1997). Our findings correlate with classical liver toxicology parameters on an individual level and could be used to support decision making in the early drug development phases.

While the identification of single m/z features in untargeted, mass spectrometry-based experiments is currently the bottleneck in metabolomics research, explorative *omic* approaches have the unique ability to shed light on mechanistic relations, as showcased by the toxicity investigations in this study, which revealed distinct elevated bile acids as likely cause of DILI. Further efforts to establish causal links between unbiased metabolomics methods and disease phenotypes will be crucial for increasing our understanding of disease pathology in the future.

## Data Availability Statement

The original contributions presented in the study are included in the article/Supplementary Material, further inquiries can be directed to the corresponding author/s.

## Ethics Statement

Ethical review and approval was not required for the animal study because all applicable international, national, and/or institutional guidelines for the care and use of animals were followed.

## Author Contributions

HD, SB, and RL conceived and designed the experiments. HD and D-AB performed the experiments, analyzed the data, and wrote the paper. RL and SB contributed reagents/materials/analysis tools. All authors contributed to the article and approved the submitted version.

## Conflict of Interest

HD, SB, and RL are employed by Boehringer Ingelheim Pharma GmbH. KG, Biberach, Germany. D-AB is employed by Boehringer Ingelheim RCV GmbH & Co KG, Vienna, Austria.

## Abbreviations

7-HOCA: 7α-Hydroxy-3-oxo-4-cholestenoic acid
ALP: Alkaline phosphatase
ALT: Alanine-aminotransferase
AST: Aspartate-aminotransferase
BSEP: Bile salt export pump
CA: Cholic acid
CCS: Collisional cross-section
CDCA: Chenodeoxycholic acid
CDCA-24G: Chenodeoxycholic acid-24 glucuronide
CYP27A1: Sterol 27-hydroxylase
CYP7A1: Cholesterol 7-alpha-monooxygenase
DCA: Deoxycholic acid
DDI: Drug-drug interaction
DILI: Drug-induced liver injury
FFAR1: Free fatty acid receptor 1
GCA: Glycocholic acid
GCDCA-S: Glycochenodeoxycholic acid sulfate
GDCA: Glycodeoxycholic acid
GPR40: G-protein-coupled receptor 40
GGT: Gamma-glutamyltransferase
GLDH: Glutamate dehydrogenase
HDCA: Hyodeoxycholic acid
NTCP: Sodium-taurocholate cotransporting polypeptide
OAT: Organic anion transporter
OATP1B1: Organic anion transporting polypeptide 1B1
OATP1B3: Organic anion transporting polypeptide 1B3
OPLS-DA: Orthogonal partial least squares discriminant analysis
TDCA: Taurodeoxycholic acid
TCA: Taurocholic acid
TOF-MS: Time-of-flight mass spectrometry
UDCA: Ursodeoxycholic acid
UPLC-TWIMS-HRMS: Ultra performance–traveling wave ion mobility separation–high-resolution mass spectrometry.
