# Supplementary material for: Untargeted Metabolomic Analysis Combined With Multivariate Statistics Reveal Distinct Metabolic Changes in GPR40 Agonist-Treated Animals Related to Bile Acid Metabolism
Source: Front Mol Biosci. 2021 Jan 15;7:598369. doi: 10.3389/fmolb.2020.598369 (PMC7843463; doi:10.3389/fmolb.2020.598369)
Supplement: Supplementary file 1 [file Data_Sheet_1.PDF]

Untargeted metabolomic analysis combined with multivariate statistics reveal distinct metabolic changes in GPR40 agonists-treated animals related to bile acid metabolism

Hannes Doerfler[1*], Dana-Adriana Botesteanu[2], Stefan Blech[1] and Ralf Laux[1]

[1]Department of Drug Metabolism & Pharmacokinetics, Boehringer Ingelheim Pharma GmbH & Co. KG, Biberach (Riß), Germany

[2]Department of Drug Discovery Sciences, Boehringer Ingelheim RCV GmbH & Co KG, Vienna, Austria

*Address for correspondence:

Dr. Hannes Doerfler, Boehringer Ingelheim Pharma GmbH & Co. KG, Drug Metabolism & Pharmacokinetics, Biberach (Riß), Germany. E-Mail: hannes.doerfler@gmx.at

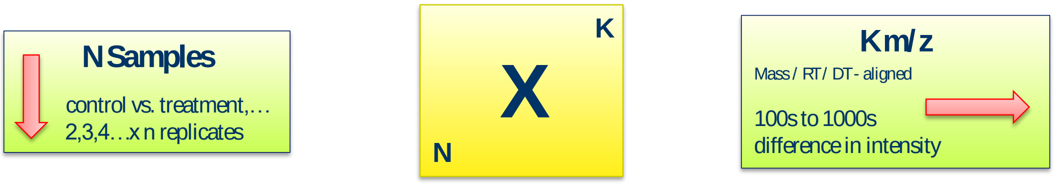

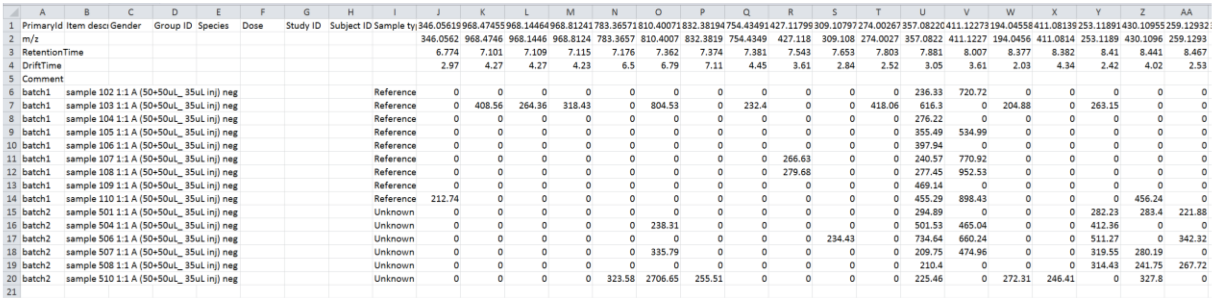

	A	B	C	D	E	F	G	H	I	J	K	L	M	N	O	P	Q	R	S	T	U	V	W	X	Y	Z	AA
1	PrimaryId	Item desc	Gender	Group ID	Species	Dose	Study ID	Subject ID	Sample ty	346.05619	968.47455	968.14464	968.81241	783.36571	810.40071	832.38194	754.43491	427.11799	309.10797	274.00267	357.08220	411.12273	194.04558	411.08139	253.11891	430.10955	259.12932
2	m/z									346.0562	968.4746	968.1446	968.8124	783.3657	810.4007	832.3819	754.4349	427.118	309.108	274.0027	357.0822	411.1227	194.0456	411.0814	253.1189	430.1096	259.1293
3	RetentionTime									6.774	7.101	7.109	7.115	7.176	7.362	7.374	7.381	7.543	7.653	7.803	7.881	8.007	8.377	8.382	8.41	8.441	8.467
4	DriftTime									2.97	4.27	4.27	4.23	6.5	6.79	7.11	4.45	3.61	2.84	2.52	3.05	3.61	2.03	4.34	2.42	4.02	2.53
5	Comment																										
6	batch1	sample 102 1:1 A (50+50uL_ 35uL inj) neg							Reference	0	0	0	0	0	0	0	0	0	0	0	236.33	720.72	0	0	0	0	0
7	batch1	sample 103 1:1 A (50+50uL_ 35uL inj) neg							Reference	0	408.56	264.36	318.43	0	804.53	0	232.4	0	0	418.06	616.3	0	204.88	0	263.15	0	0
8	batch1	sample 104 1:1 A (50+50uL_ 35uL inj) neg							Reference	0	0	0	0	0	0	0	0	0	0	0	276.22	0	0	0	0	0	0
9	batch1	sample 105 1:1 A (50+50uL_ 35uL inj) neg							Reference	0	0	0	0	0	0	0	0	0	0	0	355.49	534.99	0	0	0	0	0
10	batch1	sample 106 1:1 A (50+50uL_ 35uL inj) neg							Reference	0	0	0	0	0	0	0	0	0	0	0	397.94	0	0	0	0	0	0
11	batch1	sample 107 1:1 A (50+50uL_ 35uL inj) neg							Reference	0	0	0	0	0	0	0	0	266.63	0	0	240.57	770.92	0	0	0	0	0
12	batch1	sample 108 1:1 A (50+50uL_ 35uL inj) neg							Reference	0	0	0	0	0	0	0	0	279.68	0	0	277.45	952.53	0	0	0	0	0
13	batch1	sample 109 1:1 A (50+50uL_ 35uL inj) neg							Reference	0	0	0	0	0	0	0	0	0	0	0	469.14	0	0	0	0	0	0
14	batch1	sample 110 1:1 A (50+50uL_ 35uL inj) neg							Reference	212.74	0	0	0	0	0	0	0	0	0	0	455.29	898.43	0	0	0	456.24	0
15	batch2	sample 501 1:1 A (50+50uL_ 35uL inj) neg							Unknown	0	0	0	0	0	0	0	0	0	0	0	294.89	0	0	0	282.23	283.4	221.88
16	batch2	sample 504 1:1 A (50+50uL_ 35uL inj) neg							Unknown	0	0	0	0	0	238.31	0	0	0	0	0	501.53	465.04	0	0	412.36	0	0
17	batch2	sample 506 1:1 A (50+50uL_ 35uL inj) neg							Unknown	0	0	0	0	0	0	0	0	0	234.43	0	734.64	660.24	0	0	511.27	0	342.32
18	batch2	sample 507 1:1 A (50+50uL_ 35uL inj) neg							Unknown	0	0	0	0	0	335.79	0	0	0	0	0	209.75	474.96	0	0	319.55	280.19	0
19	batch2	sample 508 1:1 A (50+50uL_ 35uL inj) neg							Unknown	0	0	0	0	0	0	0	0	0	0	0	210.4	0	0	0	314.43	241.75	267.72
20	batch2	sample 510 1:1 A (50+50uL_ 35uL inj) neg							Unknown	0	0	0	0	323.58	2706.65	255.51	0	0	0	0	225.46	0	272.31	246.41	0	327.8	0
21																											

Supplementary Figure 1. A typical data sheet as example of a megavariate problem. The variables K (m/z features, in columns) are in excess compared to the observations, N (rows). In our case, the variables are singly charged precursor ions aligned by retention time (RT) and drift time (DT); one observation corresponds to one LC-MS run of a biological replicate.

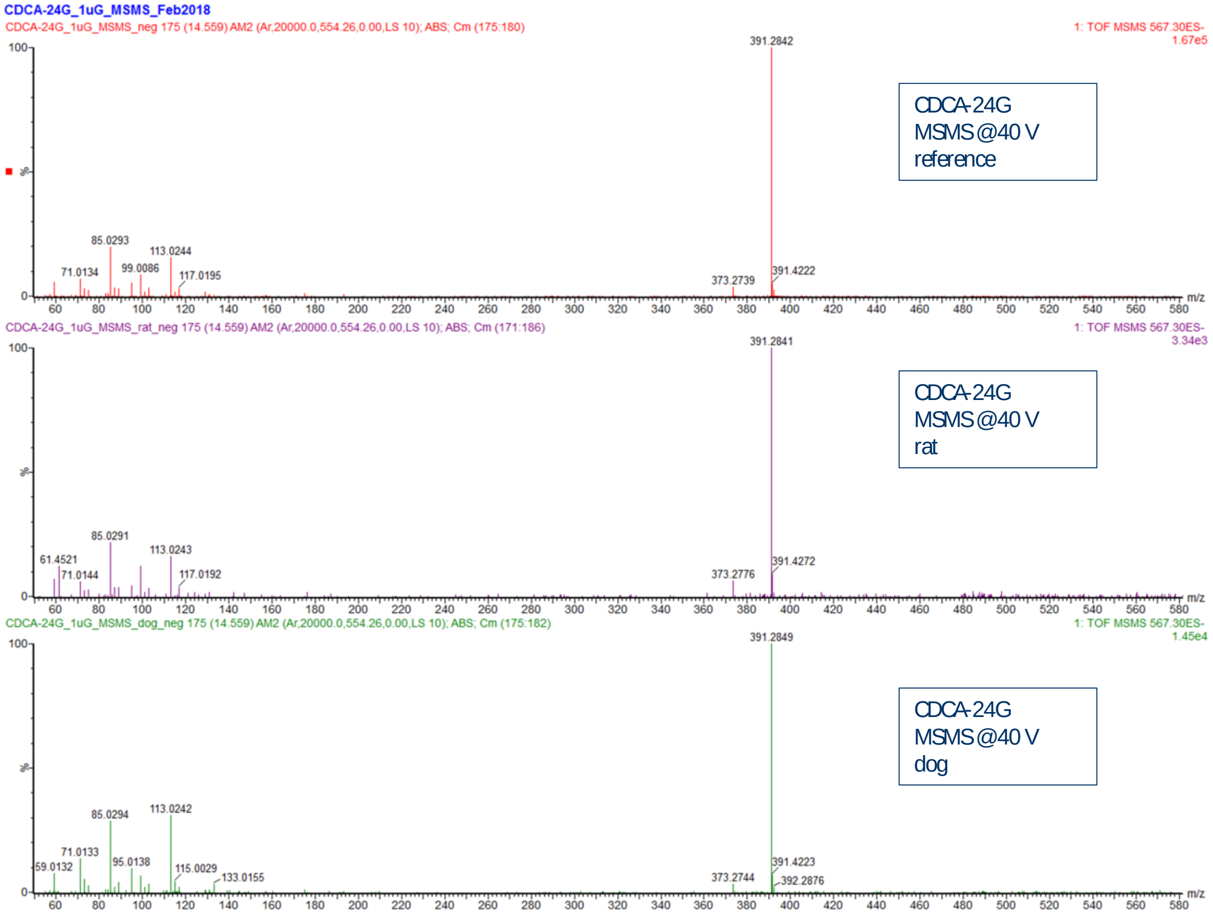

Supplementary Figure 2. MS/MS data of CDCA-24G: reference vs. signal in rat and dog

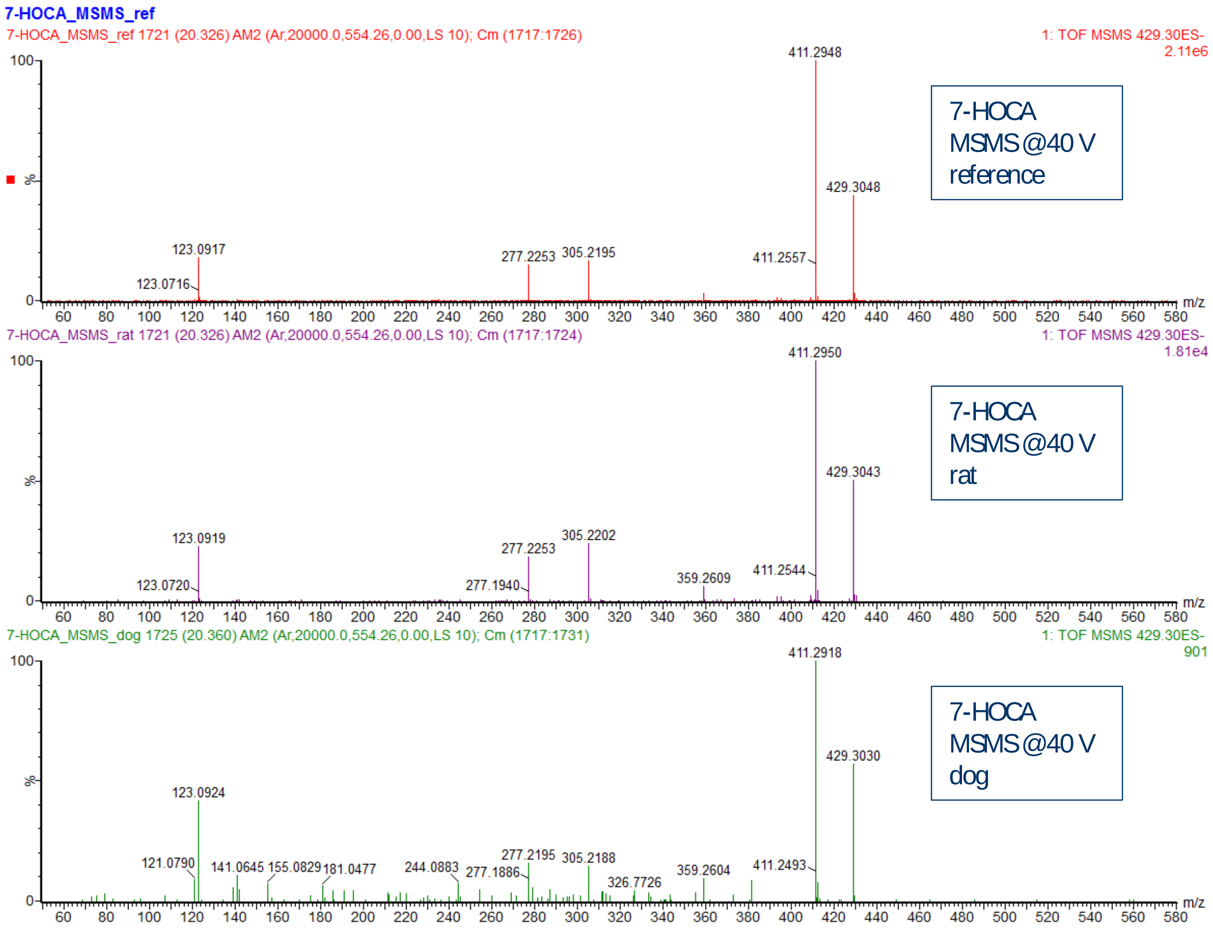

Supplementary Figure 3. MS/MS data of 7-HOCA: reference vs. signal in rat and dog

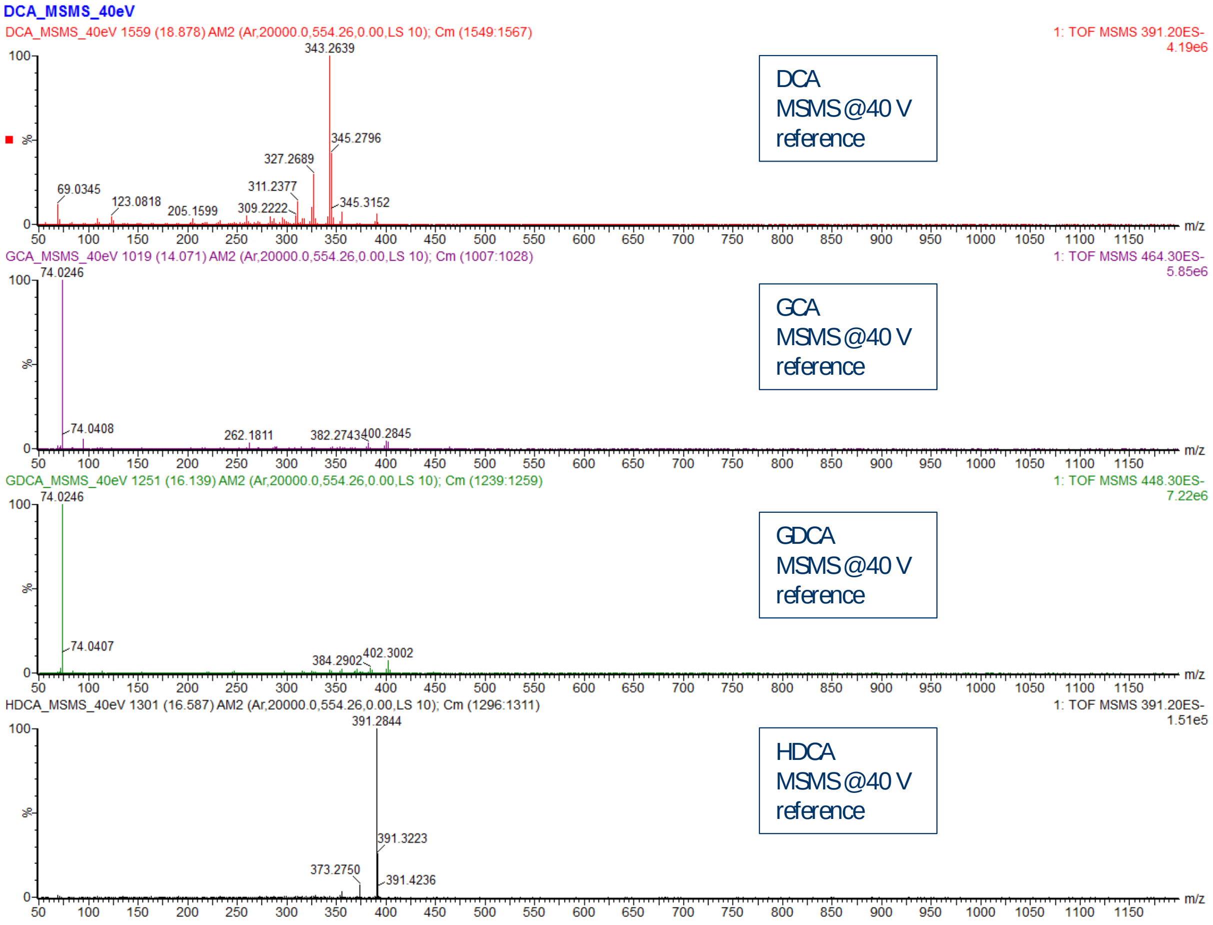

Supplementary Figure 4. MS/MS data of DCA, GCA, GDCA and HDCA (see Table 1)

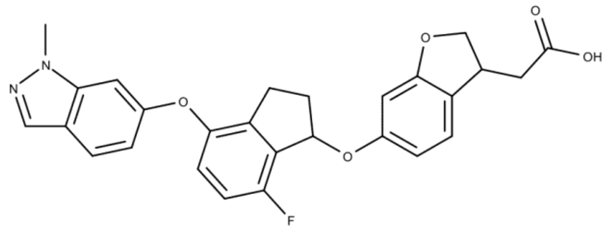

BI-1

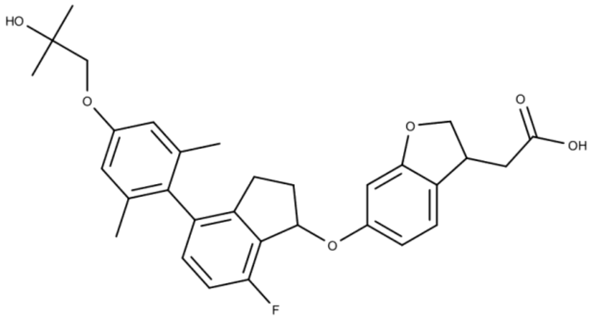

BI-2

Supplementary Figure 5. Structures of BI-1 and BI-2

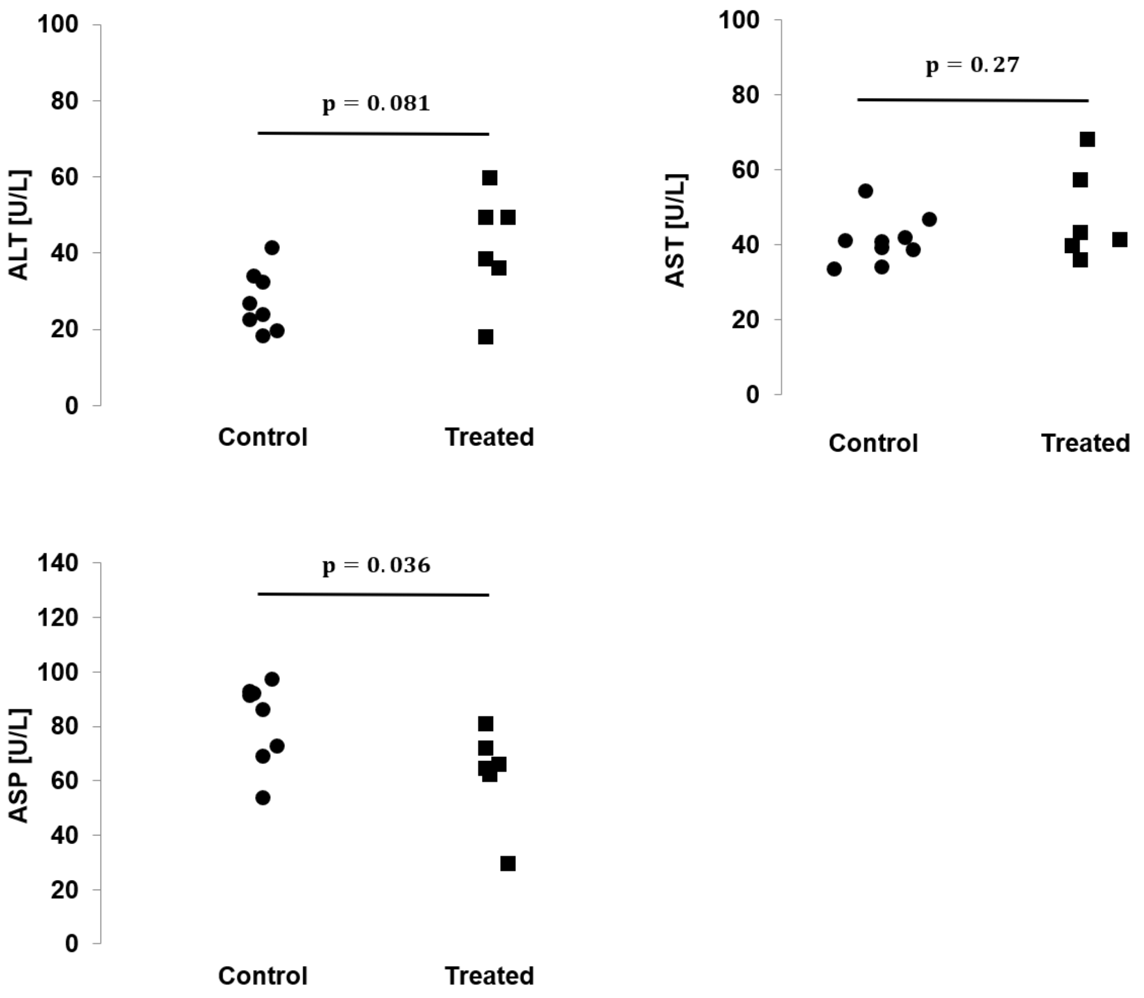

Supplementary Figure 6. ALT (panel A), AST (panel B) and ASP (panel C) enzyme levels in the control (N = 9) and treated (N = 6) mouse samples
